# Supplementary material for: Systemic Dendrimer-Peptide Therapies for Wet Age-Related Macular Degeneration
Source: Pharmaceutics. 2023 Oct 5;15(10):2428. doi: 10.3390/pharmaceutics15102428 (PMC10609940; doi:10.3390/pharmaceutics15102428)
Supplement: Supplementary file 1 [file pharmaceutics-15-02428-s001.zip › pharmaceutics-2596203-supplementary.pdf]

Supplementary Material

Systemic dendrimer-peptide therapies for wet age-related macular degeneration

Tony Wu[1], Chang Liu[1] and Rangaramanujam M. Kannan[1,*]

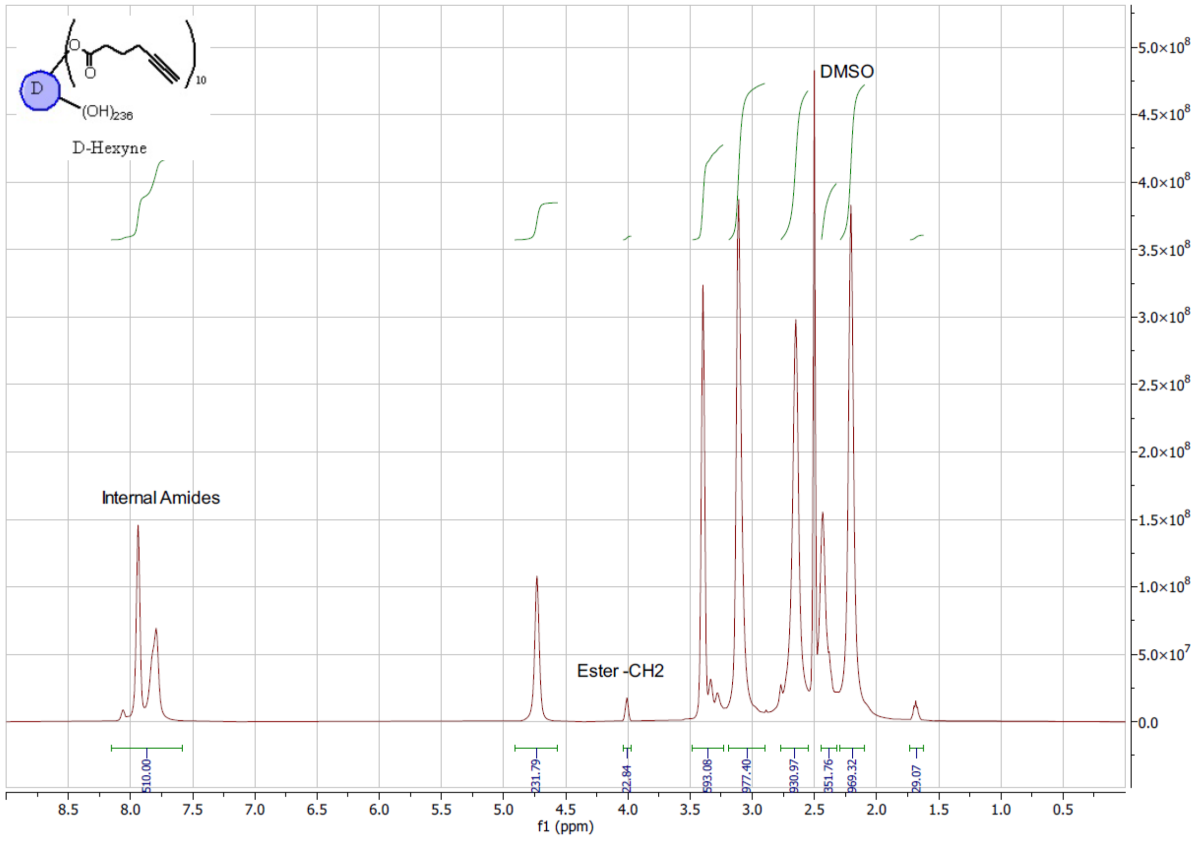

Figure S1. ^{1}H NMR spectrum of compound **1** in DMSO-*d*$_6$.

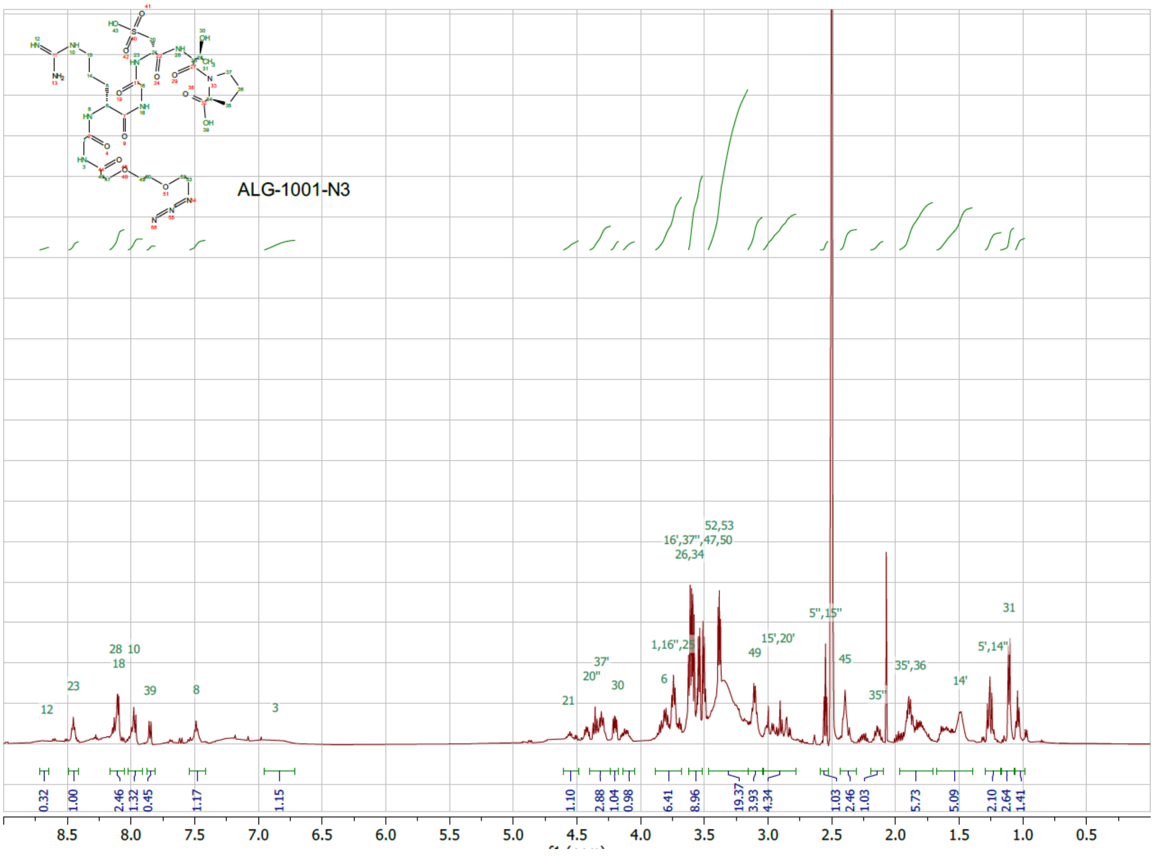

Figure S2. ^{1}H NMR spectrum of ALG-1001 in DMSO-d_6.

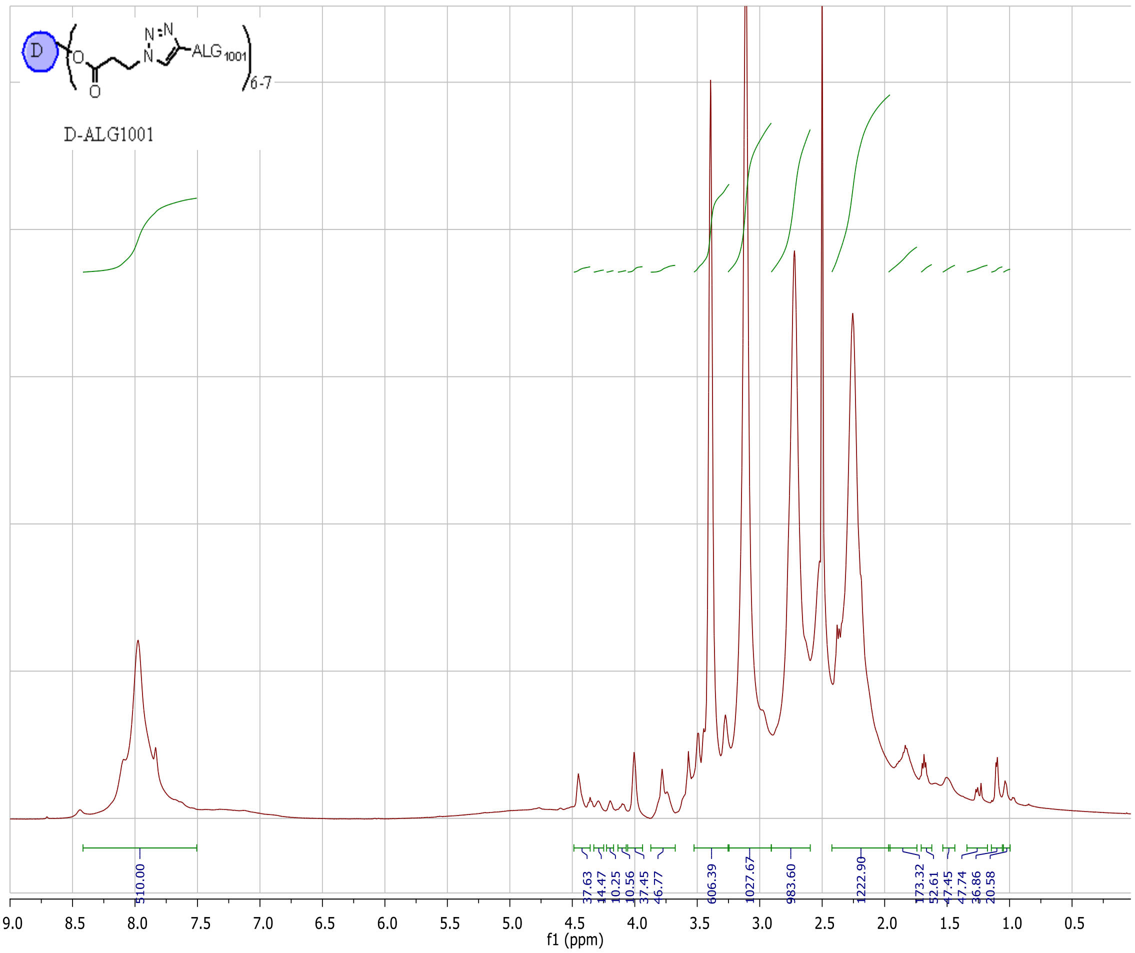

Figure S3. ^{1}H NMR spectrum of compound **3** in DMSO-*d*6.

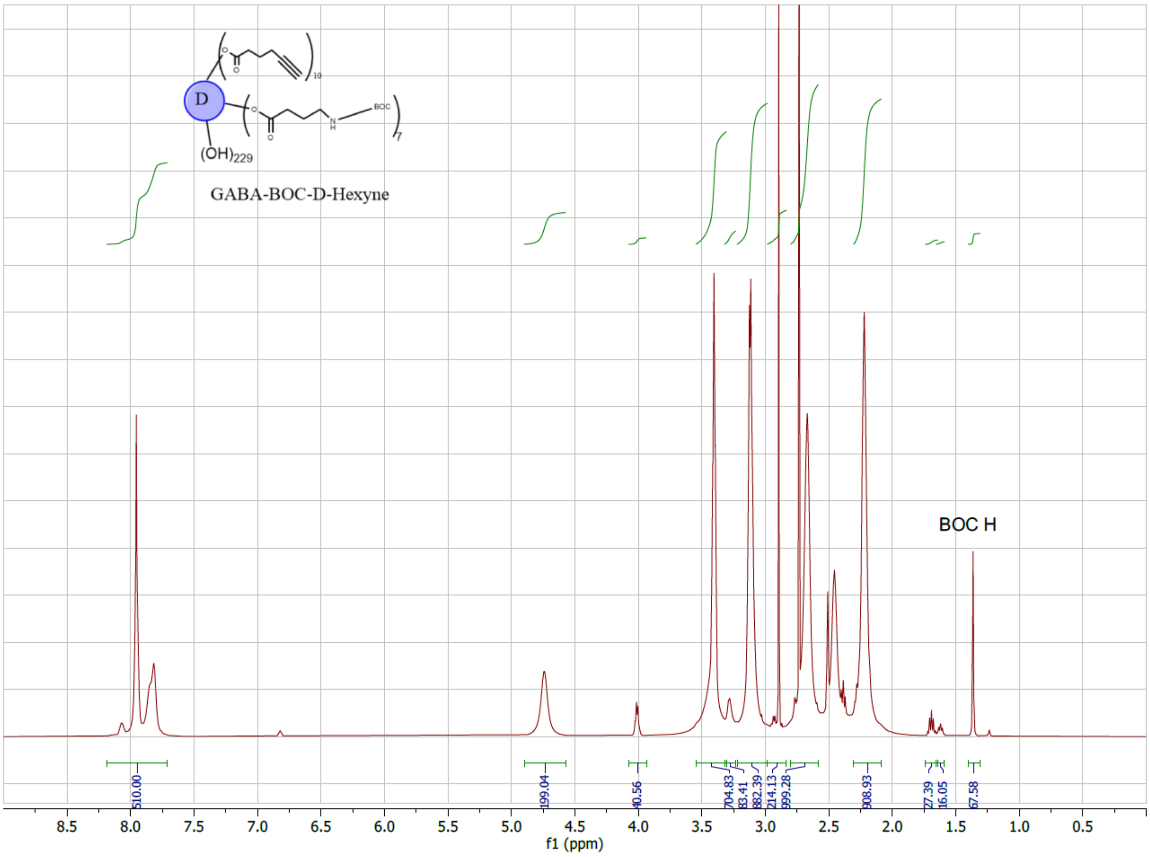

Figure S4. ^{1}H NMR spectrum of compound **4** in DMSO-d_6.

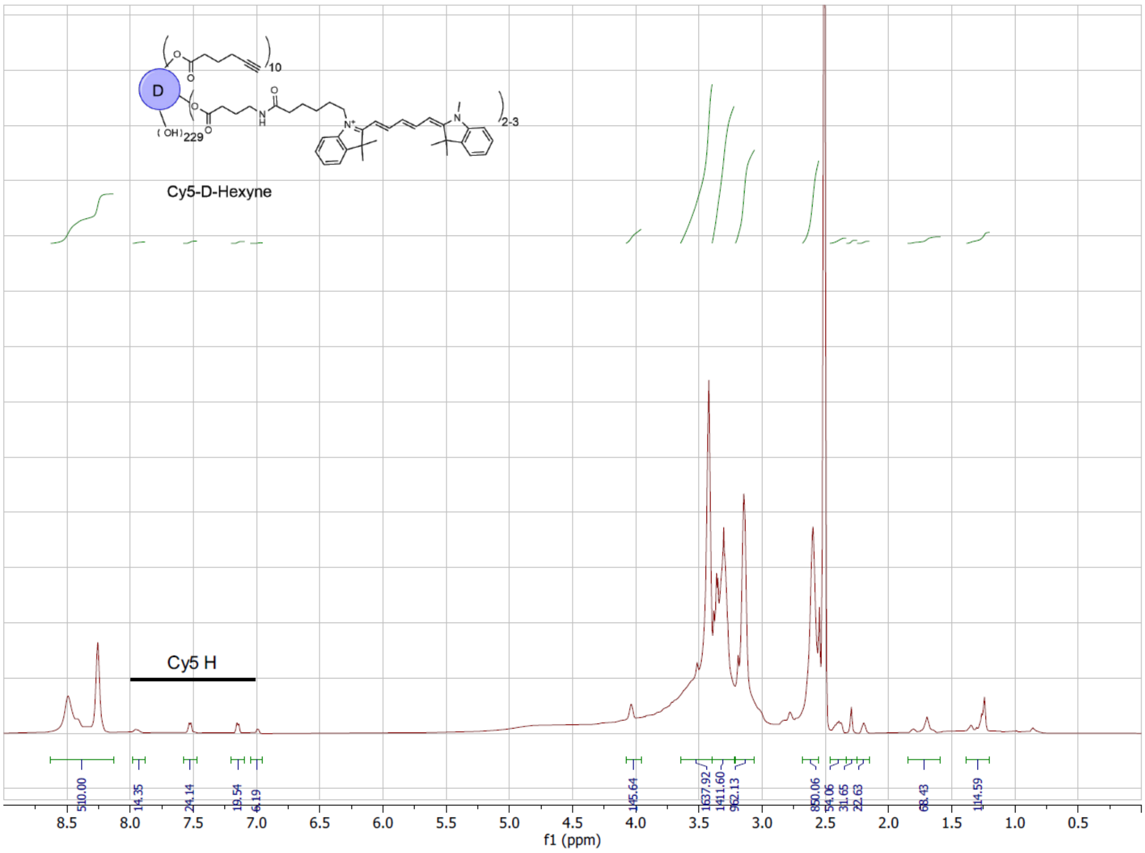

Figure S5. ^{1}H NMR spectrum of compound **5** in DMSO-d_6.

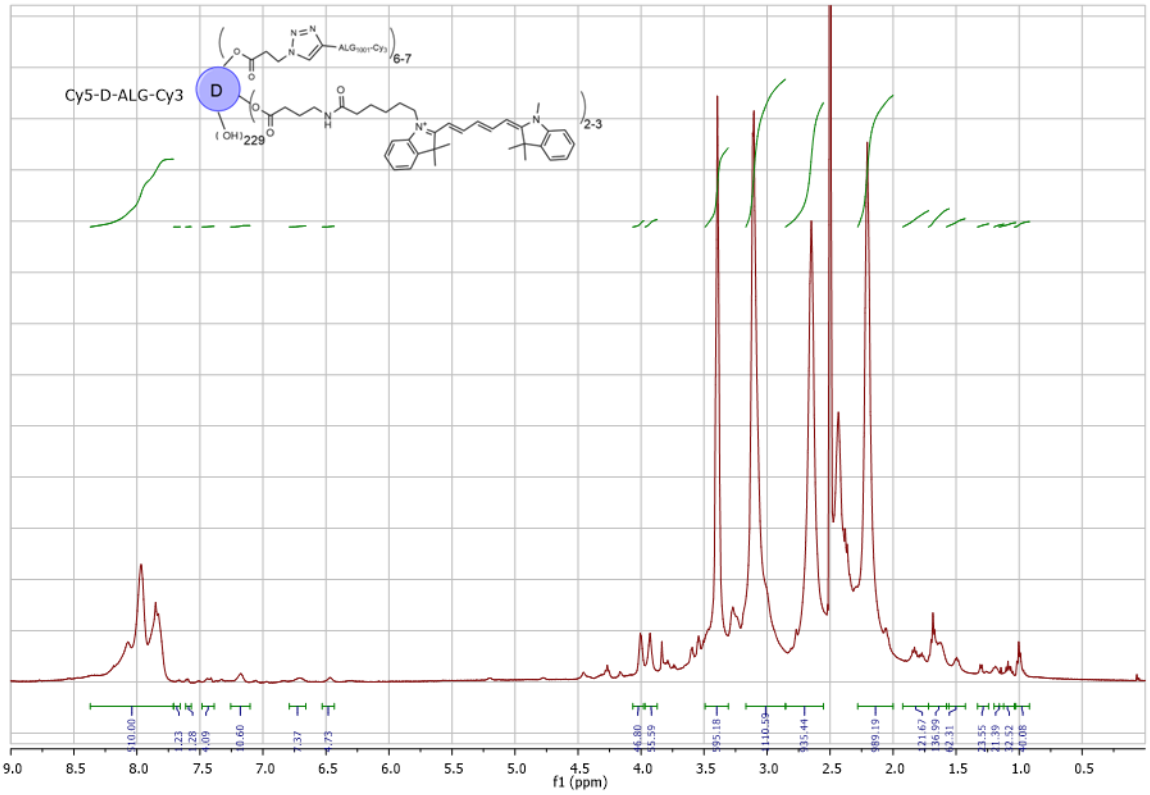

Figure S6. ^{1}H NMR spectrum of compound **6** in DMSO-d_6.

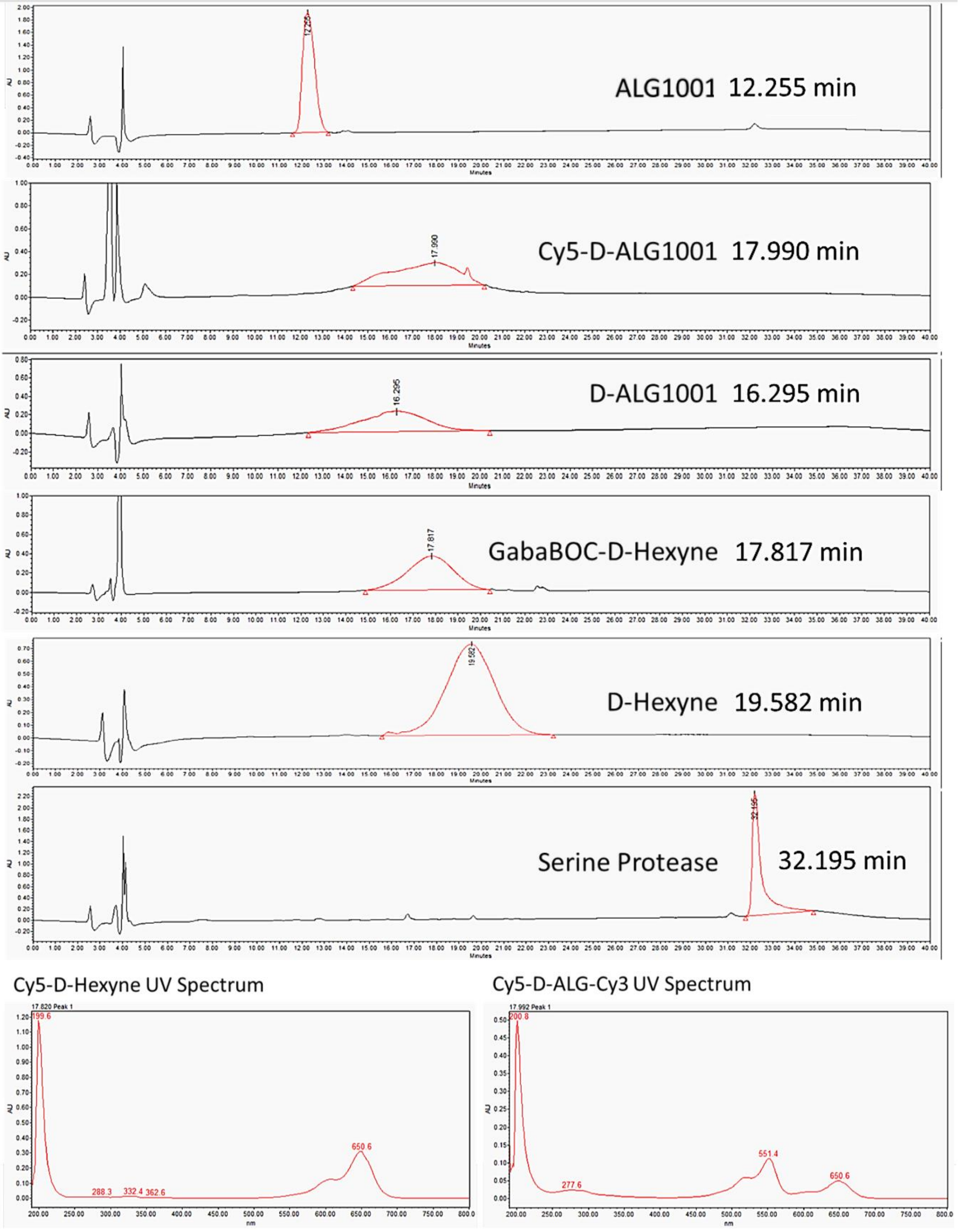

Figure S7. HPLC chromatograph of dendrimer intermediates, dendrimer-ALG conjugates, ALG-1001 peptide, and proteinase K (serine protease). Conjugation of ALG-1001 peptide to the dendrimer result in a ~3 minute-shift in retention time whereas the Cy5-labeled dendrimer-ALG1001 conjugate

did not result in a minimal shift of 0.1 minutes compared to the precursor Cy5-labeled dendrimer. To confirm peptide attachment, UV spectrum of fluorescently labeled compounds were extracted with photodiode array detector. The Cy5-labeled precursor had peaks at 200 and 650 nm which correspond to dendrimer and Cy5 peaks respectively. The Cy5-labeled dendrimer-ALG-1001 conjugate had peaks at 200, 550, and 650 nm which correspond to dendrimer, Cy3, and Cy5 respectively and indicated successful conjugation of the Cy3-labeled peptide to the Cy5-labeled dendrimer.

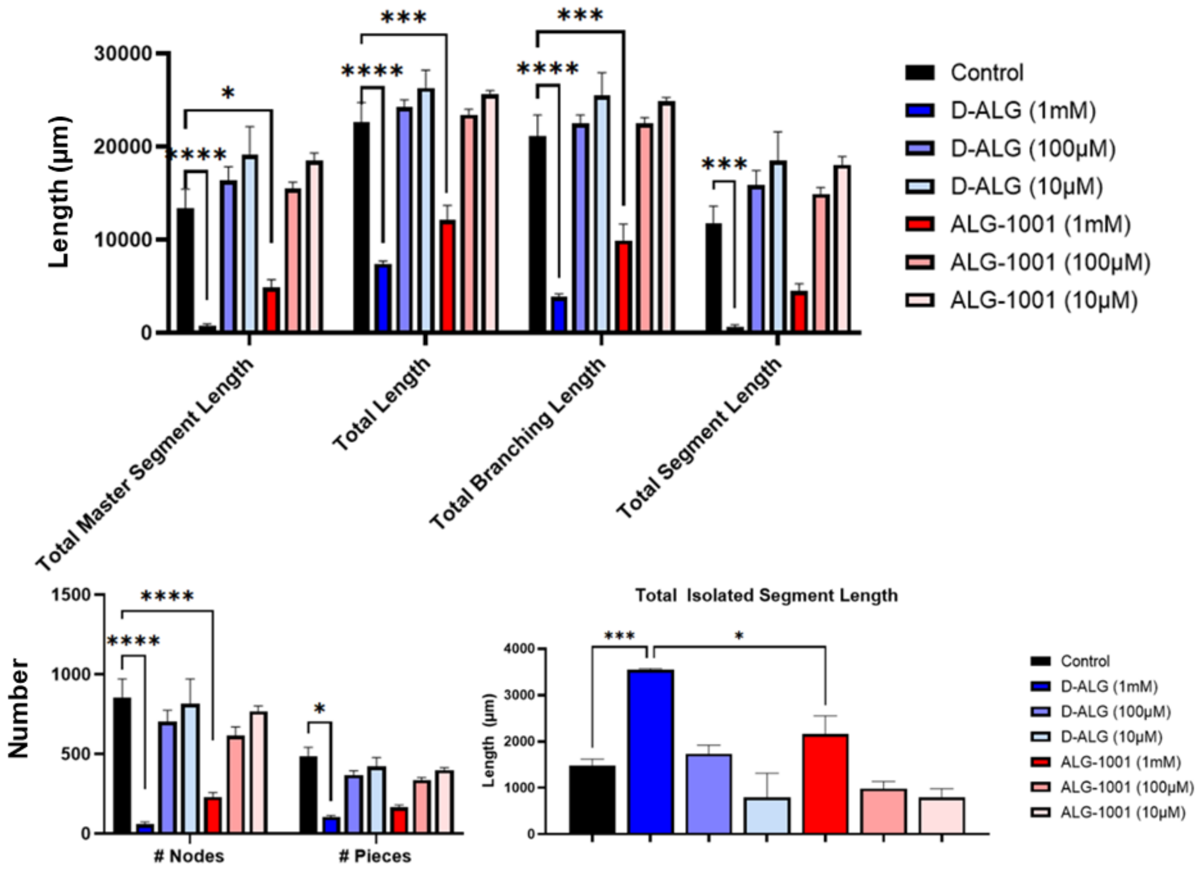

Figure S8. Quantification of vessel formation through image network analysis**.** Treatment with D-ALG and ALG-1001 at high doses result in statistically significant disruptions in vessel formation as measured through number of nodes, total length, and total isolated length. * $p < 0.05$, *** $p < 0.001$, and **** $p < 0.0001$

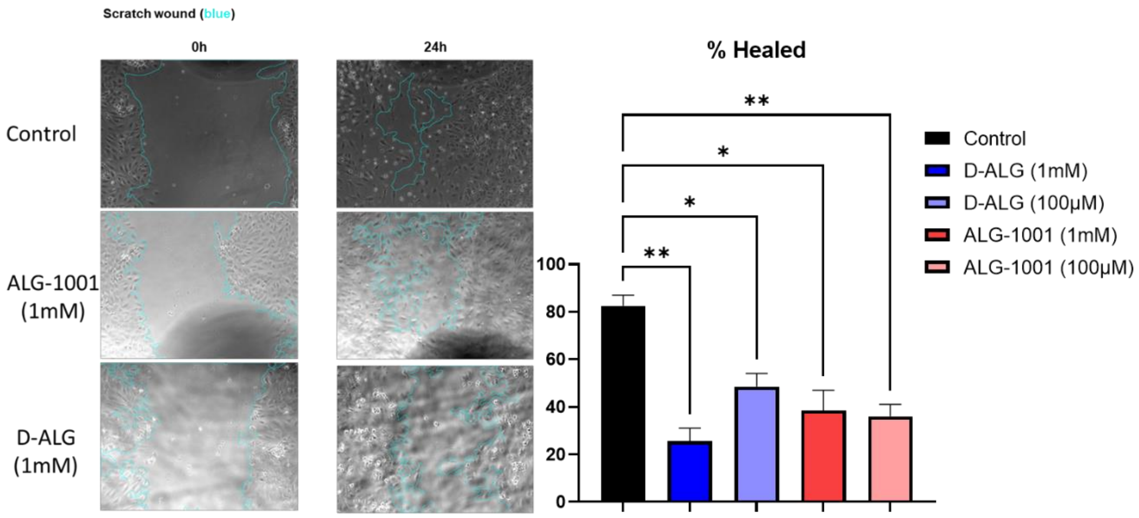

Figure S9. Quantification of *in vitro* wound healing assay. Growth of cell monolayer was analyzed 0 and 24 h after a scratch was introduced. Resultant change in monolayer area was normalized to the starting scratch area. Treatment with ALG-1001 and D-ALG result in statistically significant decrease in healing/proliferation rate of cells compared to the control. * $p < 0.05$, ** $p < 0.01$

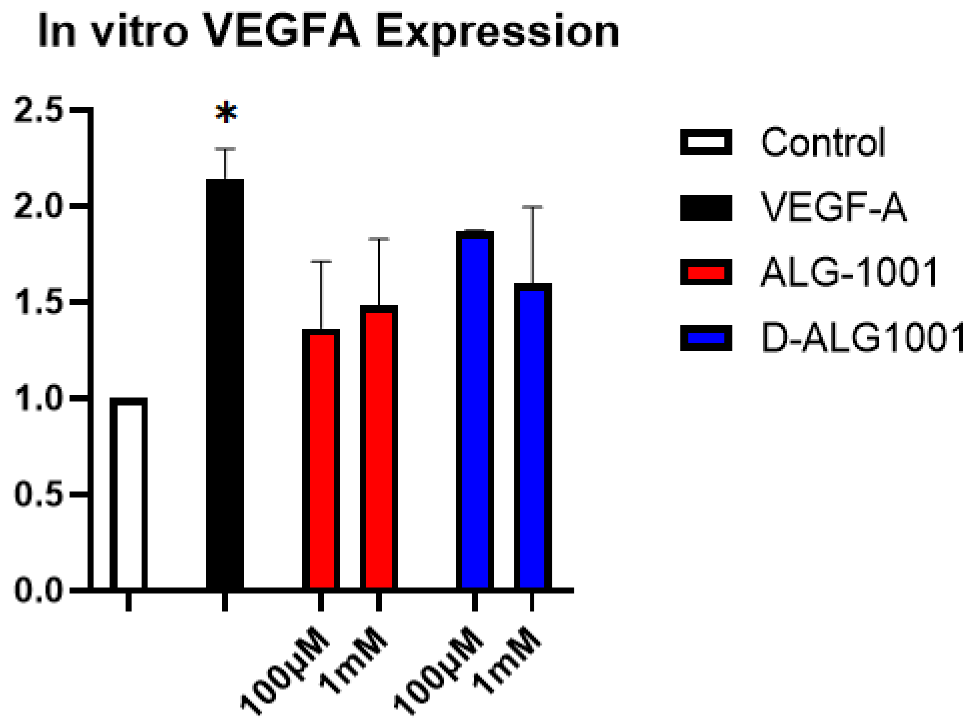

Figure S10. Expression of VEGF-A by human umbilical vein endothelial cells (HUVEC) was measured through qPCR. Statistical significance denote difference to the unstimulated and untreated cells (control). Stimulation with LPS induced a statistically significant increase in VEGF-A expression and treatment with ALG-1001 or D-ALG1001 attenuated the increase. * $p < 0.05$

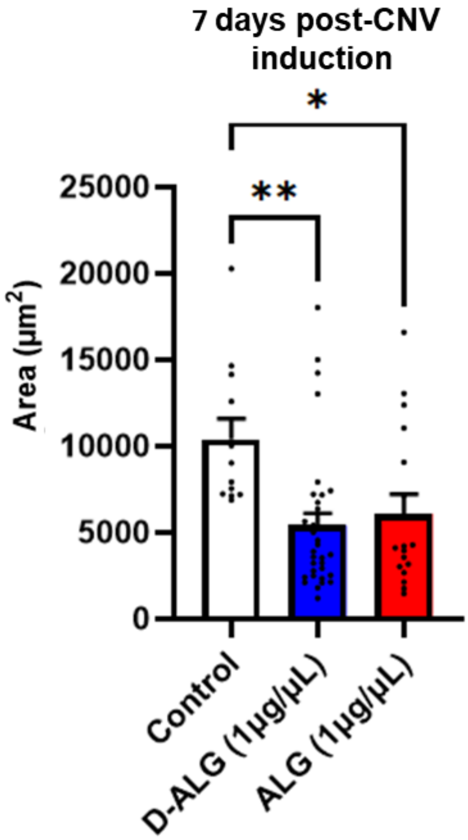

Figure S11. *In vivo* inhibition of CNV formation via intravitreal injection. CNV area quantified using ImageJ. Dendrimer conjugation preserves the *in vivo* activity of ALG-1001 peptide when administered locally (intravitreally). * $p < 0.05$, ** $p < 0.01$
